# Treatment of advanced pancreatic cancer with the long-acting somatostatin analogue lanreotide: in vitro and in vivo results

**DOI:** 10.1038/sj.bjc.6690084

**Published:** 1999-02

**Authors:** M Raderer, G Hamilton, A Kurtaran, J Valencak, I Haberl, O Hoffmann, G V Kornek, F Vorbeck, M H L Hejna, I Virgolini, W Scheithauer

**Affiliations:** Department of Internal Medicine I, Division of Oncology, University of Vienna, Vienna, Austria; Ludwig Boltzmann Institute of Clinical Oncology, KH Lainz, Vienna, Austria; Department of Nuclear Medicine, University of Vienna, Vienna, Austria; Department of Experimental Surgery, University of Vienna, Vienna, Austria; Department of Pharmacology and Toxicology, University of Vienna, Vienna, Austria; Department of Radiology, University of Vienna, Vienna, Austria

**Keywords:** lanreotide, pancreatic cancer, somatostatin receptors

## Abstract

Fourteen patients with metastatic pancreatic adenocarcinoma were treated with the long-acting somatostatin (SST) analogue lanreotide. No objective response was obtained, and the median survival was 4 months (range 1.8–7 months). Pancreatic cancer could not be visualized by means of SST-receptor (R) scintigraphy in our patients. In vitro data also demonstrated absence of SSTR2 expression, suggesting pancreatic cancer not to be a potential target for treatment with SST analogues. © 1999 Cancer Research Campaign


*British Journal of Cancer* (1999) **79**(3/4), 535–537
© 1999 Cancer Research Campaign
Article no. bjoc.1998.0084

### Treatment of advanced pancreatic cancer with the long-acting somatostatin analogue lanreotide: in vitro and in vivo results

**M Raderer[1], G Hamilton[2], A Kurtaran[3], J Valencak[1], I Haberl[4], O Hoffmann[5], GV Kornek[1], F Vorbeck[6], MHL Hejna[1], I Virgolini[3] and W Scheithauer[1]**

[1]Department of Internal Medicine I, Division of Oncology, University of Vienna, Austria; [2]Ludwig Boltzmann Institute of Clinical Oncology, KH Lainz, Vienna, Austria; [3]Department of Nuclear Medicine, [4]Department of Experimental Surgery, [5]Department of Pharmacology and Toxicology, and [6]Department of Radiology, University of Vienna, Austria

**Summary** Fourteen patients with metastatic pancreatic adenocarcinoma were treated with the long-acting somatostatin (SST) analogue lanreotide. No objective response was obtained, and the median survival was 4 months (range 1.8–7 months). Pancreatic cancer could not be visualized by means of SST-receptor (R) scintigraphy in our patients. In vitro data also demonstrated absence of SSTR2 expression, suggesting pancreatic cancer not to be a potential target for treatment with SST analogues.

**Keywords:** lanreotide; pancreatic cancer; somatostatin receptors

Pancreatic cancer is among the leading causes of cancer-related death worldwide (National Cancer Institute, 1991), and the diagnosis is established at an advanced stage in the majority of patients who are, therefore, beyond the scope of curative intervention (American Cancer Society, 1991; Warshaw and Fernandez-del Castillo, 1992). Although a positive impact of chemotherapeutic intervention upon survival and quality of life in cancer of the exocrine pancreas has been reported (Glimelius et al, 1996), chemotherapy is usually limited to patients with a good performance status and normal liver and renal functions.

According to recent data (Cascinu et al, 1995), the application of somatostatin (SST) analogues seems to prolong survival in patients suffering from various gastrointestinal cancers. Among the patients randomized between treatment with the SST analogue octreotide (OCT) and best supportive care, there was a high percentage of subjects with pancreatic cancer, who demonstrated a statistically significant benefit in terms of survival compared with untreated controls. Apart from OCT, other long-acting SST analogues have been synthesized and investigated in vitro and in vivo (Anthony et al, 1993; Kuhn et al, 1994). Lanreotide (LAN) has been evaluated for the treatment of acromegalic patients (Caron et al, 1993; Marek et al, 1994) and has been found to be well tolerated and biologically active when administered by intramuscular injection in a slow-release formulation, necessitating reinjection of the peptide at intervals of only 10–14 days.

Taken together, these findings prompted us to investigate the therapeutic potential of LAN for treatment of advanced pancreatic cancer. In addition, we have also investigated in vivo targeting of SST receptors by means of [$^{111}$In]DTPA-D-Phe$^{1}$-OCT scintigraphy ([$^{111}$In]OCT), which is being used for imaging of neuroendocrine malignancies (Krenning et al, 1993). This was accompanied by experiments to further define in vitro SSTR expression and binding of SST analogues to human tumour specimens.

*Received 2 March 1998*
*Revised 27 May 1998*
*Accepted 8 July 1998*

*Correspondence to:* M Raderer, Department of Internal Medicine I, Division of Oncology, University of Vienna, Waehringer Guertel 18–20, A-1090 Vienna, Austria

### PATIENTS AND METHODS

Fourteen untreated patients (eight women/six men) with a median age of 55 years (range 41–75 years) were enrolled in this trial. The median WHO performance status was 1 (range 0–2), and nine patients had documented weight loss exceeding 5 kg before initiation of therapy. All patients gave informed consent according to institutional guidelines. All patients had histologically verified metastatic cancer of the pancreas with at least one bidimensionally measurable target lesion as seen on computerized tomography (CT) not older than 4 weeks upon study entry. Twelve patients had liver metastases, and two had lymph node metastases. Patients with severe concomitant illness or disease metastatic to the CNS were excluded from the trial. Serial CT scanning and evaluation of tumour markers were performed every 8 weeks. In addition, the patients' performance status, weight and intake of analgesic medication as well as subjective perception of pain using a visual analogue scale were recorded before the start of treatment and before each injection of LAN.

For treatment of patients included in this study, the slow-release formulation of LAN (Somatuline LP 30-mg vials, Ipsen Biotech, Paris, France) was used, given by deep intramuscular injection every 10 days until documented progression or the patients' wish to withdraw from the study. The minimal time span between OCT scanning and the first injection of LAN had to be at least 14 days.

All patients underwent scanning by means of $^{111}$In-labelled OCT, using the commercially available preparation (OctreoScan, Mallinckrodt Medical, UK). For imaging purposes, 10 μg DTPA-D-Phe$^{1}$-OCT were labelled with 120–150 MBq of $^{111}InCl_3$, according to the manufacturer's description. Patients were given the tracer by intravenous bolus injection before initiation of therapy, and scanning was performed according to published standard methods (Krenning et al, 1993).

### Human tumour samples

Pancreatic cancer cells cultivated from ascitic fluid ($n = 3$) and primary pancreatic adenocarcinomas ($n = 5$) or obtained during surgery ($n = 3$) or autopsy ($n = 2$) were investigated for the presence of SSTR2 mRNA by means of Northern blotting. In addition, binding studies using both $^{111}$In-labelled OCT and LAN according to published methods (Scatchard, 1949; Virgoloni et al, 1994) were carried out in tumour samples and short-term cultures obtained from ascitic fluid.

### RESULTS

All patients entered in the study received treatment with LAN as scheduled. A median number of eight injections were administered (range 5–14); four patients had stable disease (SD) lasting between 3.5 and >6 months, whereas the remaining ten patients progressed during treatment.

Four patients showed a reduction in CA19-9 values by more than 30% for 4–16 weeks, and two of these patients also displayed an improvement in WHO performance status lasting for 8 and 10 weeks respectively. No beneficial influence of treatment on body weight could be demonstrated, and the intake of analgesic medication as well as subjective perception of pain did not improve compared with baseline evaluations.

At the time of this evaluation, all patients have died except one, who is alive with ongoing stable disease 6 months after initiation of therapy. The median time to progression was 2.0 months (range 1.5–6.0 months) and the median survival was 4.0 months (range 1.8–7.0 months).

No severe side-effects necessitating discontinuation of treatment were experienced by our patients, and only four patients had mild gastrointestinal complaints consisting of meteorism accompanied by mild diarrhoea in three patients and mild nausea in two. One additional patient developed discrete swelling and erythema at the injection site.

All 14 patients underwent scanning with [$^{111}$In]OCT before initiation of LAN treatment. No focal tracer uptake corresponding to tumour sites could be demonstrated in primary tumours, lymph nodes or liver metastases by means of [$^{111}$In]OCT. Northern blotting did not demonstrate the presence of mRNA specific for SSTR2 in human samples, and no significant binding of both [$^{111}$In]OCT and [$^{111}$I]LAN to tumour samples could be demonstrated.

### DISCUSSION

Despite the recent demonstration of a positive influence of chemotherapy on survival and quality of life in patients with pancreatic cancer (Glimelius et al, 1996), the overall prognosis for individuals with advanced disease remains dismal. As a result of the limited efficacy of treatment, there is continuing interest in attempts to identify new therapeutic options. Based on in vitro data suggesting the potential of SST analogues to provide an anti-mitogenic stimulus in various tumour models (Qin et al, 1992; Weckbecker et al, 1992; Reubi et al, 1994), Cascinu et al (1995) have reported a beneficial impact of treatment with OCT in patients with advanced pancreatic cancer. In addition, a therapeutic benefit for patients treated with high-dose OCT (Ebert et al, 1994) was reported in another study, implicating SST analogues as potential tools for treatment of advanced pancreatic cancer.

In this study, we have investigated the application of the long-acting SST analogue LAN in patients with pancreatic cancer. In contrast to OCT, which has a serum half-life of approximately 100 min after subcutaneous injection (Bauer et al, 1982), LAN has been demonstrated to be biologically active and well tolerated when given as a slow-release formulation (Marek et al, 1994; Caron et al, 1995) every 10–14 days at the dose applied in our series. Therapeutic results in our study, however, were disappointing: four patients had SD lasting between 3.5 and 6 months, whereas the remaining patients rapidly progressed. The median survival was 4 months, and a short improvement in performance status could be demonstrated in only two patients. Analgesic intake, subjective pain perception and body weight were not beneficially influenced in our patients. These results are consistent with phase II data reported with the use of OCT, in which median survivals between 2 and 6 months have been observed (Kiljn et al, 1990; Canobbio et al, 1992; Hughier et al, 1992; Ebert et al, 1994). In contrast, Cascinu et al (1995) reported a significantly prolonged survival in patients suffering from pancreatic cancer with the administration of OCT, with the median survival being 15 weeks compared with 7 weeks in untreated patients. Although the survival time of treated patients seems to be comparable with our data and other trials reported (Kiljn et al, 1990; Canobbio et al, 1992; Hughier et al, 1992; Ebert et al, 1994), the survival in the control cohort appears to be exceptionally short, offering a plausible explanation for the statistically significant difference.

Because of conflicting in vitro and in vivo data with regard to SST receptor expression and growth inhibition in various cancers (Reubi et al, 1988, 1990; Janson et al, 1996), we have attempted to define the in vivo receptor status of our patients by means of [$^{111}$In]DTPA-D-Phe$^{1}$-OCT, which is widely applied for detection and staging of neuroendocrine tumours (Krenning et al, 1993). Scintigraphy was performed before application of LAN to avoid a potential influence on receptors after prolonged peptide administration, and thus to prevent false scan results. Both OCT and LAN have been reported to bind to at least one of the same SST receptor subtypes with high affinity, namely SSTR2 (Viollet et al, 1995; Janson et al, 1996). In our patients, no positive [$^{111}$In]OCT scans could be obtained, indicating the absence of clinically relevant amounts of SSTR2. This is in accordance with data obtained in our tumour samples and with results published by various groups (Reubi et al, 1988; Krenning et al, 1993; Taylor et al, 1994; Buscail et al, 1996), indicating the loss of expression of SSTR2 in pancreatic cancer. Because this receptor subtype seems to play a crucial role in the growth inhibition attributed to SST analogues, the absence of SSTR2 offers the most likely explanation for the disappointing therapeutic results. Taken together, our results add to the accumulating body of evidence that SST analogues do not seem to result in convincing palliation or prolongation of survival in advanced pancreatic cancer.

### ACKNOWLEDGEMENTS

This study was supported in part by a grant of the Österreichische Nationalbank (Jubiläumsfonds), grant no. 6587. The authors would like to thank JR Bauer, Sabine Bergmann and Tatjana Traub for assistance.

*© Cancer Research Campaign 1999*

### REFERENCES

American Cancer Society (1991) *Cancer Facts and Figures 1991*. American Cancer Society: Atlanta

Anthony L, Johnson D, Hande K, Shaff M, Winn S, Krozely M and Oates J (1993) Somatostatin analogue phase I trials in neuroendocrine neoplasms. *Acta Oncol* **32**: 217–223

Bauer W, Briner U, Doepfner W, Haller R, Huguenin R, Marbach P, Petcher TH and Pless J (1982) SMS 201-995: a very potent and selective octapeptide analogue of somatostatin with prolonged action. *Life Sci* **31**: 1133–1140

Buscail L, Saint-Laurent N, Chastre E, Vaillant JC, Gespach C, Capella G, Kalthoff H, Lluis F, Vaysse N and Susini C (1996) Loss of sst2 somatostatin receptor gene expression in human pancreatic and colorectal cancer. *Cancer Res* **56**: 1823–1827

Canobbio L, Boccardo F, Cannata D, Gallotti P and Epis R (1992) Treatment of advanced pancreatic carcinoma with the somatostatin analogue BIM 23014. *Cancer* **69**: 648–650

Caron P, Cogne M, Gusthiot-Joudet B, Wakim S, Catus F and Bayard F (1995) Intramuscular injections of slow release lanreotide (BIM 23014) in acromegalic patients previously treated with continuous subcutaneous infusion of octreotide (SMS 201-995). *Eur J Endocrinol* **132**: 320–325

Cascinu S, Del Ferro E and Catalano G (1995) A randomised trial of octreotide vs best supportive care only in advanced gastrointestinal cancer patients refractory to chemotherapy. *Br J Cancer* **71**: 97–101

Ebert M, Friess H, Berger HG and Büchler MW (1994) Role of octreotide in the treatment of pancreatic cancer. *Digestion* **55** (suppl. 1): 48–51

Glimelius B, Hoffman K, Sjöden PO, Jacobsson G, Sellström H, Enander LK, Linne T and Svensson C (1996) Chemotherapy improves survival and quality of life in advanced pancreatic and biliary cancer. *Ann Oncol* **7**: 593–600

Hughier M, Samama G, Testart J, Mauban S, Fingerhut A, Nassar J, Houry S, Saeck D, De Mestier I and Favre JP (1992) Treatment of adenocarcinoma of the pancreas with somatostatin and gonadoliberin (luteinizing hormone-releasing hormone). *Am J Surg* **164**: 348–353

Janson ET, Gobl A, Kalkner KM and Oberg K (1996) A comparison between the efficacy of somatostatin receptor scintigraphy and that of in situ hybridization for somatostatin receptor subtype 2 messenger RNA to predict therapeutic outcome in carcinoid patients. *Cancer Res* **56**: 2561–2565

Kiljn JGM, Hoff AM, Planting AST, Verweij J, Kok T, Lamberts SWJ, Portengen H and Foekens JA (1990) Treatment of patients with metastatic pancreatic and gastrointestinal tumours with the somatostatin analogue sandostatin: a phase II study including endocrine effects. *Br J Cancer* **62**: 627–630

Krenning EP, Kwekkeboom DJ, Bakker WH, Breeman W, Kooij P, Oei H, Van Hagen M, Postema F, De Jong M and Reubi JC (1993) Somatostatin receptor scintigraphy with $^{111}$In-DTPA-D-Phe-1 and $^{123}$I-Tyr-3-octreotide: the Rotterdam experience with more than 1000 patients. *Eur J Nucl Med* **20**: 716–731

Kuhn JM, Legrand A, Ruiz JM, Obach R, De-Ronzan J and Thomas F (1994) Pharmacokinetic and pharmacodynamic properties of a long acting formulation of the new somatostatin analogue, lanreotide, in normal healthy volunteers. *Br J Clin Pharmacol* **38**: 213–219

Marek J, Hana V, Krsek M, Justova V, Catus F and Thomas F (1994) Long-term treatment of acromegaly with the slow release somatostatin analogue lanreotide. *Eur J Endocrinol* **131**: 20–26

National Cancer Institute (1991) *Annual Cancer Statistics Review 1973–1988*. NIH publication no. 91-2789. Department of Health and Human Services: Bethesda, MD

Qin Y, Schally AV and Willems G (1992) Treatment of liver metastases of human colon cancers in nude mice with somatostatin analogue RC-160. *Int J Cancer* **52**: 791–796

Reubi JC, Horisberger U, Essed JE, Jeekel J, Klijn JGH and Lamberts SWJ (1988) Absence of somatostatin receptors in human exocrine pancreatic adenocarcinomas. *Gastroenterology* **95**: 760–763

Reubi JC, Krenning E, Lamberts SWJ and Kvols L (1990) Somatostatin receptors in malignant tissues. *Steroid Biochem Mol Biol* **37**: 1073–1077

Reubi JC, Horisberger U and Laissue J (1994) High density of somatostatin receptors in veins surrounding human cancer tissue: role in tumour–host interaction? *Int J Cancer* **56**: 681–688

Scatchard G (1949) The attraction of proteins for small molecules and ions. *Ann NY Acad Sci* **51**: 660–672

Taylor JE, Theveniau MA, Bashirzadeh R, Reisine T and Eden PA (1994) Detection of somatostatin receptor subtype 2 (SSTR2) in established tumors and tumor cell lines: evidence for SSTR2 heterogeneity. *Peptides* **15**: 1229–1236

Viollet C, Prevost G, Maubert E, Faivre-Bauman A, Gardette R, Kordon C, Loudes C, Slama A and Epelbaum J (1995) Molecular pharmacology of somatostatin receptors. *Fundam Clin Pharmacol* **9**: 107–113

Virgolini I, Yang Q, Li S, Angelberger P, Neumold N, Niederle B, Scheithauer W and Valent P (1994) Cross-competition between vasoactive intestinal peptide (VIP) and somatostatin for binding to tumor cell receptors. *Cancer Res* **54**: 690–700

Warshaw AL and Fernandez-del Castillo C (1992) Pancreatic carcinoma. *N Engl J Med* **326**: 455–465

Weckbecker G, Liu R, Tolcsvai L and Bruns C (1992) Antiproliferative effects of the somatostatin analogue octreotide (SMS 201-995) on ZR-75-1 human breast cancer cells in vivo and in vitro. *Cancer Res* **52**: 4973–4978

*© Cancer Research Campaign 1999*
